# SVM Classifier – a comprehensive java interface for support vector machine classification of microarray data

**DOI:** 10.1186/1471-2105-7-S4-S25

**Published:** 2006-12-12

**Authors:** Mehdi Pirooznia, Youping Deng

**Affiliations:** 1 Department of Biological Sciences, University of Southern Mississippi, Hattiesburg, Mississippi 39406, USA

## Abstract

**Motivation:**

Graphical user interface (GUI) software promotes novelty by allowing users to extend the functionality. SVM Classifier is a cross-platform graphical application that handles very large datasets well. The purpose of this study is to create a GUI application that allows SVM users to perform SVM training, classification and prediction.

**Results:**

The GUI provides user-friendly access to state-of-the-art SVM methods embodied in the LIBSVM implementation of Support Vector Machine. We implemented the java interface using standard swing libraries.

We used a sample data from a breast cancer study for testing classification accuracy. We achieved 100% accuracy in classification among the BRCA1–BRCA2 samples with RBF kernel of SVM.

**Conclusion:**

We have developed a java GUI application that allows SVM users to perform SVM training, classification and prediction. We have demonstrated that support vector machines can accurately classify genes into functional categories based upon expression data from DNA microarray hybridization experiments. Among the different kernel functions that we examined, the SVM that uses a radial basis kernel function provides the best performance.

The SVM Classifier is available at .

## Background

High-density DNA microarray measures the activities of several thousand genes simultaneously and the gene expression profiles have been recently used for the cancer and also other disease classification. The Support Vector Machine (SVM) [1,2] is a supervised learning algorithm, useful for recognizing subtle patterns in complex datasets. It is one of the classification methods successfully applied to the diagnosis and prognosis problems. The algorithm performs discriminative classification, learning by example to predict the classifications of previously unclassified data. The Support Vector Machine (SVM) was one of the methods successfully applied to the cancer diagnosis problem in the previous studies [3,4]. In principle, the SVM can be applied to very high dimensional data without altering its formulation. Such capacity is well suited to the microarray data structure.

The popularity of the SVM algorithm comes from four factors [5]. 1) The SVM algorithm has a strong theoretical foundation, based on the ideas of VC (Vapnik Chervonenkis) dimension and structural risk minimization [2]. 2) The SVM algorithm scales well to relatively large datasets. 3) The SVM algorithm is flexible due in part to the robustness of the algorithm itself, and in part to the parameterization of the SVM via a broad class of functions, called kernel functions. The behavior of the SVM can be modified to incorporate prior knowledge of a classification task simply by modifying the underlying kernel function. 4) Accuracy: The most important explanation for the popularity of the SVM algorithm is its accuracy. The underlying theory suggests explanations for the SVMs excellent learning performance, its widespread application is due in large part to the empirical success the algorithm has achieved [5].

## Implementation

We have developed a simple graphical interface to our implementation of the SVM algorithm, called SVM Classifier. This interface allows novice users to download the software for local installation and easily apply a sophisticated machine learning algorithm to their data. We implemented a publicly accessible application that allows SVM users to perform SVM training, classification and prediction. For details on using the software, sample dataset and explanations of the underlying algorithms, we refer readers to the web site and the references listed there. SVM users might also be interested in a number of other licensed SVM implementations that have been described previously, including LIBSVM [6].

**Figure 1:**
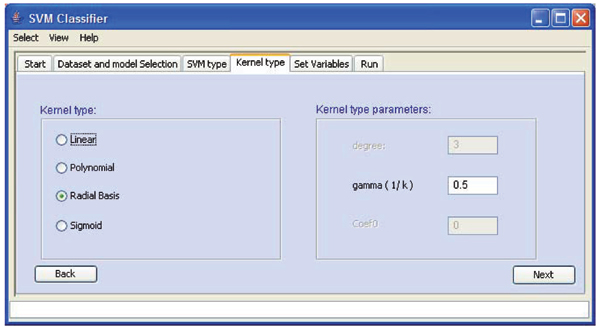
SVM Classifier interface screen shot.

We used the SVM algorithms implemented by the Libsvm team [6], as a core. In order to maximize cross-platform compatibility SVM Classifier is implemented in java using standard swing libraries (Figure 1).

The open source, cross-platform Apache Ant, and free edition of Borland JBuilder 2005 Foundation are used as the build tools. Although developed on WinXP OS, SVM Classifier has been successfully tested on Linux and other Windows platforms, and will run on Mac OS9 with the Swing extension. Users are able to run SVM Classifier on any computer with java 1.4 runtime or higher version.

**Figure 2:**
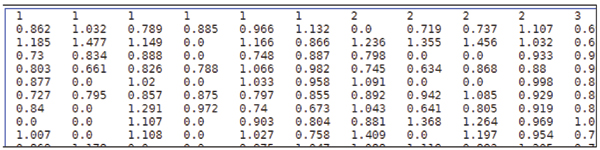
**Labelled Data File Format Screenshot**. The format of training and testing data file is: <label> <index1>:<value1> <index2>:<value2> ... <label> is the target value of the training data. For classification, it should be an integer which identifies a class (multi-class classification is supported). For regression, it's any real number. For one-class SVM, it's not used so can be any number. <index> is an integer starting from 1, <value> is a real number. The indices must be in an ascending order. The labels in the testing data file are only used to calculate accuracy or error. If they are unknown, just fill this column with a number.

**Figure 3:**
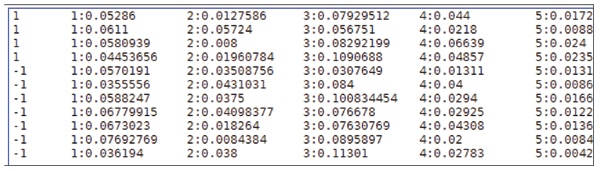
**Delimited File Format Screenshot**. The delimited File Format is a common format for the microarray experiment. It can be extracted from most microarray experiments by using any spreadsheet. The format of training and testing data file is: <label> <label> <label> ... <value1> <value2> <value3> ... <label> is the target value of the training data. For classification, it should be an integer which identifies a class (multi-class classification is supported). For regression, it's any real number. For one-class SVM, it's not used so can be any number. <value> is a real number. The indices must be in an ascending order. The labels in the testing data file are only used to calculate accuracy or error. If they are unknown, just fill this column with a number.

The application has two frames, the classification and the prediction frame. In both frames data file format can be imported either as a "Labeled" or as a "Delimited" data file format (Figure 2 and Figure 3).

In the classification frame user will create a model from the training dataset for classification (C-SVC, nu-SVC), regression (epsilon-SVR, nu-SVR), and distribution estimation.

In this frame user is able to import the training dataset into the application, select the path to save the model file, select the appropriate SVM and kernel type and create a model for the dataset. The model file can be later used for the prediction purpose. There is also a choice for cross validation. Cross validation (CV) technique is used to estimate the accuracy of each parameter combination in the specified range and helps us to decide the best parameters for classification problem.

**Figure 4:**
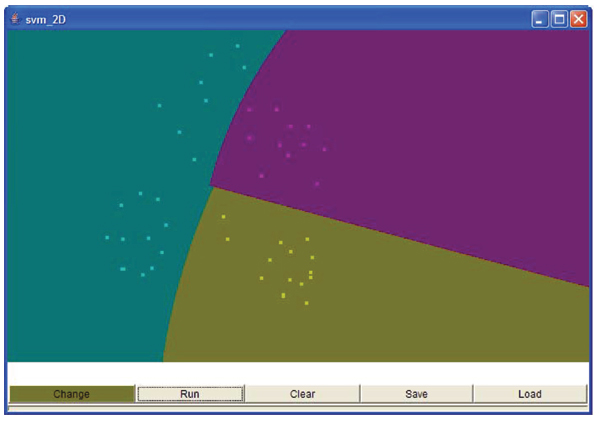
Two Dimensional Data Visualising Screenshot.

In prediction frame the model will be applied to the test data to predict the classification of unknown data. We have also provided a tool for viewing the two dimensional data that can be accessed from the view menu bar (Figure 4).

### Kernel Types

*K*(*x*_*i*_, *x*_*j*_) = Φ(*x*_*i*_)^*T*^Φ(*x*_*j*_) is called the kernel function. Here training vectors *x*_*i *_are mapped into a higher (probably infinite) dimensional space by the function Φ. There are following four basic kernels: linear, polynomial, radial basic function (RBF), and sigmoid:

1. Linear: *K*(*x*_*i*_, *x*_*j*_) = *x*_*i*_^*T*^*x*_*j*_

2. Polynomial: The polynomial kernel of degree d is of the form

*K*(*x*_*i*_, *x*_*j*_) = (*x*_*i*_, *x*_*j*_)^*d*^

3. RBF: The Gaussian kernel, known also as the radial basis function, is of the form



Where *σ *stands for a window width

4. Sigmoid: The sigmoid kernel is of the form

*K*(*x*_*i*_, *x*_*j*_) = tanh(*k*(*x*_*i*_*x*_*j*_) + *ϑ*)

When the sigmoid kernel is used with the SVM one can regard it as a two-layer neural network.

### SVM Types

#### 1. C-SVC: C-Support Vector Classification (Binary Case)

Given a training set of instance-label pairs (*x*_*i*_, *y*_*i*_), i = 1,...,*l *where *x*_*i *_∈ *R*^*n *^and *y *∈ {1, -1}^*l*^, ←the support vector machines (SVM) require the solution of the following optimization problem:



SVM finds a linear separating hyperplane with the maximal margin in this higher dimensional space. C > 0 is the penalty parameter of the error term [7,2]. The decision function is:



#### 2. nu-SVC: *ν*-Support Vector Classification (Binary Case)

The parameter *ν *∈ (0, 1) is an upper bound on the fraction of training errors and a lower bound of the fraction of support vectors [8]. Given training vectors *x*_*i *_∈ *R*^*n*^, i = 1,...,l, in two classes, and a vector *y *∈ *R*^*l *^such that *y*_*i *_∈ {1, -1}, the primal form considered is:



And the decision function is:



#### 3. epsilon-SVR: *ε*-Support Vector Regression (*ε*-SVR)

One extension of the SVM is that for the regression task. A regression problem is given whenever Y = ℝ for the training data set Z = {(*x*_*i*_, *y*_*i*_) ∈ *X *× *Y *| i = 1,...,M} and our interest is to find a function of the form *f*: X → RD. The primal formulation for the SVR is then given by:



We have to introduce two types of slack-variables *ξ*_*i *_and , one to control the error induced by observations that are larger than the upper bound of the *ε*-tube, and the other for the observations smaller than the lower bound. The approximate function is:



#### 4. nu-SVR: *ν*-Support Vector Regression (*ν*-SVR)

Similar to *ν*-SVC, for regression, [8,9] use a parameter *ν *to control the number of support vectors. However, unlike *ν*-SVC where C is replaced by *ν *here *ν *replaces the parameter *ε *of *ε*-SVR. Then the decision function is the same as that of *ε*-SVR.

#### 5. One-class SVM: distribution estimation

One-class classification's difference from the standard classification problem is that the training data is not identically distributed to the test data. The dataset contains two classes: one of them, the target class, is well sampled, while the other class is absent or sampled very sparsely. Schölkopf *et al*. [9] have proposed an approach in which the target class is separated from the origin by a hyperplane. The primal form considered is:



And the decision function is:



### Cross Validation

The goal of using cross validation is to identify good parameters so that the classifier can accurately predict unknown data [6].

A common way is to separate training data into two parts, one of which is considered unknown in training the classifier. Then the prediction accuracy on this set can more precisely reflect the performance on classifying unknown data. An improved version of this procedure is cross-validation.

In v-fold cross-validation, the training set is divided into v subsets of equal size. Sequentially one subset is tested using the classifier trained on the remaining v - 1 subsets. Thus, each instance of the whole training set is predicted once so the cross-validation accuracy is the percentage of data which are correctly classified. The cross-validation procedure can prevent the overfitting problem [6].

### Shrinking

Chang and Lin [6] mentioned that since for many problems the number of free support vectors (i.e. 0 <*α*_*i *_< C) is small, the shrinking technique reduces the size of the working problem without considering some bounded variables [6,10].

### Caching

Caching is another technique for reducing the computational time. Since Q (Q is an *l *by *l *positive semi definite matrix, *Q*_*ij *_= *y*_*i*_*y*_*j*_*K*(*x*_*i*_, *x*_*j*_)) is fully dense and may not be stored in the computer memory, elements Q_ij _are calculated as needed. Usually a special storage using the idea of a cache is used to store recently used Q_ij _[6,10].

## Results and Discussion

We presented an evaluation of the different classification techniques presented previously. Data from a breast cancer study [11] is used in this study. The data consists of 22 cDNA microarrays, each representing 3226 genes based on biopsy specimens of primary breast tumors of 7 patients with germ-line mutations of BRCA1, 8 patients with germ-line mutations of BRCA2, and 7 with sporadic cases. We took log2 of the data to perform the classification using the three kernels.

**Figure 5:**
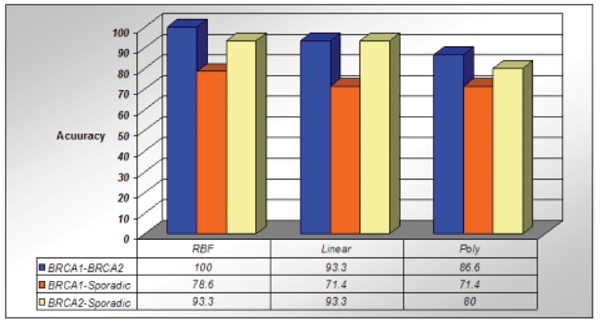
Classification accuracy shown with polynomial, linear and radial basis function kernel among the BRCA1–BRCA2, BRCA1-sporadic and BRCA2-sporadic.

We have achieved 100% accuracy in classification among the BRCA1–BRCA2 samples with RBF kernel of SVM. RBF kernel also shows better performance among all data as shown in Figure 5.

## Conclusion

We have developed a java GUI application that allows SVM users to perform SVM training, classification and prediction. We have demonstrated that support vector machines can accurately classify genes into functional categories based upon expression data from DNA microarray hybridization experiments. Among the different kernel functions that we examined, the SVM that uses a radial basis kernel function provides the best performance.

## Availability and Requirements

Project name: SVM Classifies

Project home page: 

Operating systems: platform independent

Programming language: Java Swing

Other requirements: Java JRE 1.4.2 or higher

License: GNU GPL

Any restrictions to use by non-academics: none

## List of abbreviations

VC dimension: Vapnik Chervonenkis dimension

C-SVC: C-Support Vector Classification

nu-SVC: *ν*-Support Vector Classification

nu-SVR: *ν*-Support Vector Regression (*ν*-SVR)

*ε*-SVR: *ε*-Support Vector Regression (epsilon-SVR)

## Authors' contributions

Mehdi Pirooznia has designed and implemented the study and handled java software development, software engineering issues for SVM Classifier and data set preparation and testing. Youping Deng has coordinated and directed the project and revised the manuscript. Both authors have read and approved the final manuscript.
